# MRI-based vertebral bone quality score as a novel bone status marker of patients with adolescent idiopathic scoliosis

**DOI:** 10.1038/s41598-024-63426-9

**Published:** 2024-05-31

**Authors:** Dan-dan Yang, Yi Li, Jiang-yu Tian, Ya Li, Jian Liu, Yun-song Liu, Xin-wen Cao, Chuan Liu

**Affiliations:** 1 Radiology Department, The Third People’s Hospital of Chengdu, No. 82 Qinglong Road, Qingyang District, Chengdu, 610031 Sichuan China; 2 Department of Radiology, Key Laboratory of Birth Defects and Related Diseases of Women and Children of Ministry of Education, West China Second University Hospital, Sichuan University, No. 20 Renmin South Road, Wuhou District, Chengdu, 610044 Sichuan China

**Keywords:** Adolescent idiopathic scoliosis, Vertebral bone quality, Bone mineral density, Quantitative computed tomography, Diagnostic markers, Musculoskeletal system, Magnetic resonance imaging

## Abstract

To investigate the application of MRI-based vertebral bone quality (VBQ) score in assessing bone mineral density (BMD) for patients with adolescent idiopathic scoliosis (AIS). We reviewed the data of AIS patients between January 2021 and October 2023 with MRI, whole-spine plain radiographs, quantitative computed tomography (QCT) and general information. VBQ score was calculated using T1-weighted MRI. Univariate analysis was applied to present the differences between variables of patients with normal BMD group (QCT Z-score > − 2.0) and low BMD group (QCT Z-score ≤ − 2.0). The correlation between VBQ score and QCT Z-score was analyzed with Pearson correlation test. A multivariate logistic regression model was used to determine the independent factors related to low BMD. Receiver operating characteristic curve (ROC) was drawn to analyze the diagnostic performance of VBQ score in distinguishing low BMD. A total of 136 AIS patients (mean age was 14.84 ± 2.10 years) were included, of which 41 had low BMD. The low BMD group had a significantly higher VBQ score than that in normal group (3.48 ± 0.85 vs. 2.62 ± 0.62, *P* < 0.001). The VBQ score was significantly negative correlated with QCT Z score (*r* =  − 0.454, *P* < 0.001). On multivariate analysis, VBQ score was independently associated with low BMD (OR: 4.134, 95% CI 2.136–8.000, *P* < 0.001). The area under the ROC curve indicated that the diagnostic accuracy of the VBQ score for predicting low BMD was 81%. A sensitivity of 65.9% with a specificity of 88.4% could be achieved for distinguishing low BMD by setting the VBQ score cutoff as 3.18. The novel VBQ score was a promising tool in distinguishing low BMD in patients with AIS and could be useful as opportunistic assessment for screening and complementary evaluation to QCT before surgery.

## Introduction

Adolescent idiopathic scoliosis (AIS) is the most common spinal deformity in children and adolescents aged from 10 to 18 years old^1^. It is a complex three-dimensional spinal deformity with a risk of progression and has unknown etiology^2,3^.

Surgical intervention can correct spinal deformities as much as possible, maintain overall balance of the spine, and thus maximize the long-term health of the patient's spine^4^. However, obtaining good outcomes in spinal correction surgery can be challenging for patients with poor bone quality, which is at an increased risk of postoperative complications such as instrumentation failure, screw loosening, cage subsidence and proximal junctional kyphosis^5,6^. In addition, poor bone quality is also one of the risk factors for worsening scoliotic curve^7–9^. Therefore, screening of bone mineral density (BMD) is integral to effective preoperative optimization of potentially low BMD or osteoporotic patients with AIS.

Dual-energy X-ray absorptiometry (DXA) has been widely used clinically to evaluate the bone mass due to its low radiation exposure, low cost, and strong applicability. However, DXA may incorrectly estimate the BMD in patients with severe obesity, sclerosing disease or scoliosis^10,11^. Conversely, three-dimensional scanning with quantitative computed tomography (QCT) can overcome the deficiencies of DXA by providing accurate volumetric BMD measurement but requires the higher radiation dose. In 2019, Ehresman et al.^12^ proposed a novel magnetic resonance imaging (MRI)-based vertebral bone quality (VBQ) score to evaluate BMD. The VBQ score can be used to evaluate detrimental fat infiltration within the vertebral body based on MRI T1-weighted imaging of lumbar spine. Since then, many studies have confirmed that the VBQ score was correlated with DXA T-scores, and has high predictive value for vertebral fragility fractures, making it a novel and reproducible tool for radiation-free bone density screening^12–15^. To our knowledge, to date the VBQ score has not been used to evaluate bone quality in patients with AIS.

Considering MRI is a customary preoperative assessment for AIS patients undergoing surgery, we envision whether the MRI-based VBQ score can be used for opportunistic assessment of bone density in AIS patient. Therefore, the study aimed to (1) evaluate the correlation between VBQ score and QCT-based BMD in patients with AIS, (2) investigate the diagnostic performance of VBQ score in assessing BMD.

## Methods

### Patients

Patients diagnosed with AIS between January 2021 and October 2023 were retrospectively reviewed. AIS is diagnosed by a coronal plane angle (measured by the Cobb method) of more than 10 degrees^16^, without neuromuscular, congenital, or other etiology. In the unit, patients with AIS were screened in a one-stop shop, including X-ray film, computer tomography (CT) and MRI. The inclusion criteria were: (1) age between 10 and 18 years old, (2) having QCT measurement report. The exclusion criteria were: (1) patients who received brace treatment or surgery, (2) patients who received lumbar surgical intervention due to conditions other than AIS, or those who have other diseases affecting bone metabolism, (3) incomplete medical charts, (4) with poor-quality images due to artifact. Electronic medical records for the enrolled patient were reviewed for anthropometric and radiologic data.

### QCT Scanning and BMD measurement

QCT measurements were obtained based on lumbar CT scan. CT images were analyzed using the QCT Pro v6.1 analysis software (Mindways Software, Inc.). Average BMD (mg/cm^3^) was reported. And references for vertebral BMD Z scores based on age and sex were provided by the manufacturer of the QCT software. According to the current ISCD recommendations for children and adolescent, QCT Z-score of ≤ − 2.0 was defined as low BMD a^17^. The eligible patients were categorized into normal BMD group (Z-score > -2.0) and low BMD group (Z-score ≤ − 2.0).

### VBQ score measurement and calculation

All MRI T1-weighted images were performed with either 1.5 T GE (Discovery MR 750; GE Medical Systems, Milwaukee, WI) or 3.0 T Siemens scanners (MAGNETOM Skyra, Siemens Healthcare, Erlangen, Germany). The T1-weighted acquisitions included TR (ms) = 400–850, TE (ms) = 8–20, slice thickness (mm) = 3, Matrix = 320 × 210 or 384 × 185, FOV (cm) = 240 × 240.

**Figure 1:**
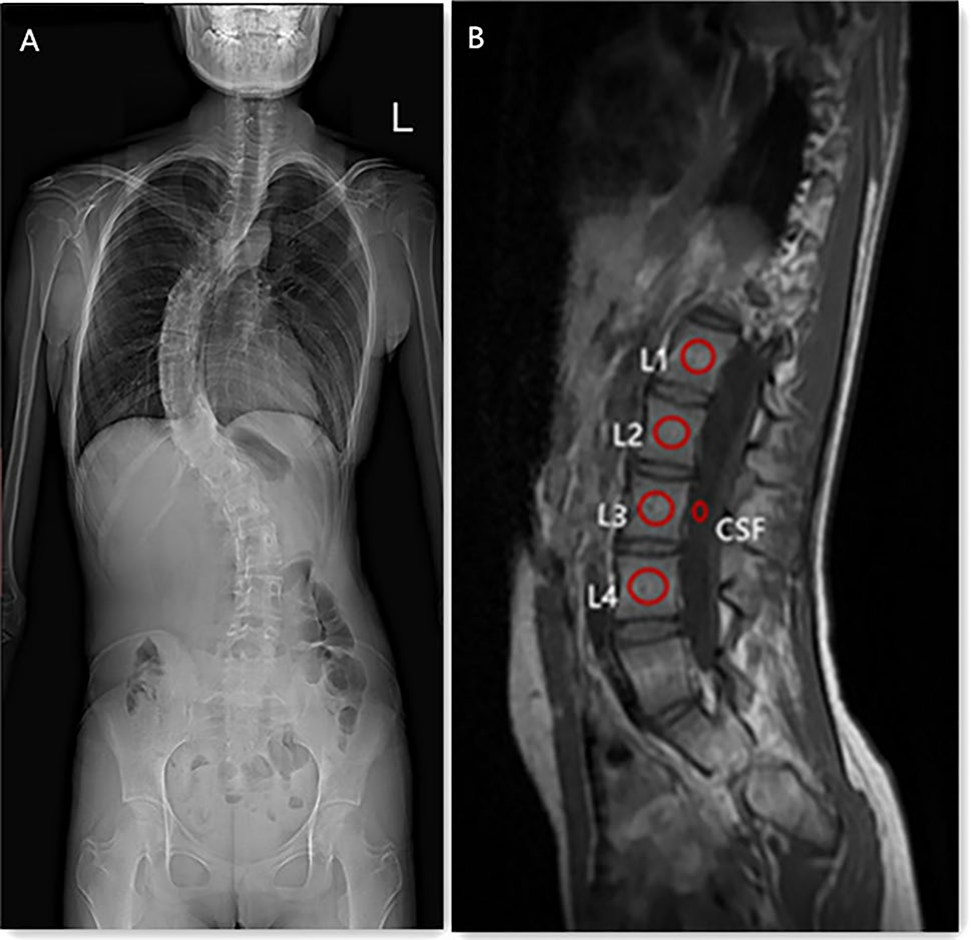
(**A**) a standing full-length posteroanterior plain radiography of an AIS patient with aged 16 years. (**B**) The signal intensity of regions of interest (circles) utilized for the computation of L1-L4 vertebral bone quality (VBQ) score on sagittal non-contrast-enhanced T1-weighted MRI.

The measurement of VBQ was based on the method described by Ehresman et al.^12^ with several changes and additions. On the midsagittal plane of lumbar spinal non-contrast-enhanced T1-weighted MRI, region of interests (ROIs) were placed in the medullary portions of L1-L4 vertebral bodies and in the cerebrospinal fluid space at the level of L3 and then the signal intensity (SI) of the trabecular bone from the vertebra L1 to L4 were automatically calculated. The placement of ROIs should avoid the cortical bone and the posterior vertebral venous plexus and cover as much of the trabecular region as possible while not requiring a consistent ROI size. When the ROI of L1-L4 cannot be obtained in one mid-sagittal slice due to severe lumbar scoliosis, the ROIs should put in the midsagittal slice for each individual vertebral body level by adjusting parasagittal slice. The measurement schematic is shown in Fig. 1. The VBQ score was calculated according to the following formula:

$$VBQ = \frac{{ SI_{{median\;\left( {L1 - L4} \right)}} }}{{SI_{{CSF_{L3} }} }}$$

The measurement was accomplished by two independent researchers in order to evaluate the interrater reliability. And to evaluate the intra-rater reliability, the first researcher repeated the measurement for 40 patients randomly picked from the sample a week after the first-round measurement. During the measurement, both researchers were blinded to the data of patients.

### Ethics approval and consent to participate

This study was approved by the institutional review board of Chengdu Third People’s Hospital. The requirement for informed consent from the study subjects was waived by the IRB of (Chengdu Third People’s Hospital of Science and Technology/Research committee) due to the retrospective study design. Only patients’ file number were extracted with the data and no names or identifiable information was included. In addition, the committee ensured that all methods used in this research was performed in accordance with relevant guidelines/regulations.

### Statistical analysis

All analyses were performed using SPSS 23.0 (IBM Corp. Armonk, New York, USA) and Graphpad prsim 9 (GRAPHPAD SOFTWARE, LLC, California, USA). Descriptive statistics constituted medians or means ± standard derivations for continuous variables and proportions for categorical variables. For continuous variables, the differences between groups were assessed using Kruskal–Wallis H test or Student's t test for statistical significance. And categorical variables were analyzed by the chi-square test and Fisher's exact test. The correlation between the VBQ score and QCT Z-score was analyzed with Pearson correlation test and linear regression. A multivariate logistic regression model was used to analyze factors independently related to low BMD, which was reported as the odds ratio (OR) with 95% confidence interval (95% CI). Variables with a *P* value < 0.1 in the univariate analysis as the relevant factors for low BMD were included in the multivariate model. The Youden index was used to determine cutoff values with best diagnostic performance via receiver operating characteristic (ROC) curve analysis, and the area under the curve (AUC) of variables was calculated. Further, to evaluate the intra-rater or inter-rater reliability, the interclass correlation coefficient (ICC) with 95% confidence interval (CI) was calculated based on data from 40 patients. A *P* value < 0.05 was considered statistically significant.

## Results

### General characteristics of study population

**Figure 2:**
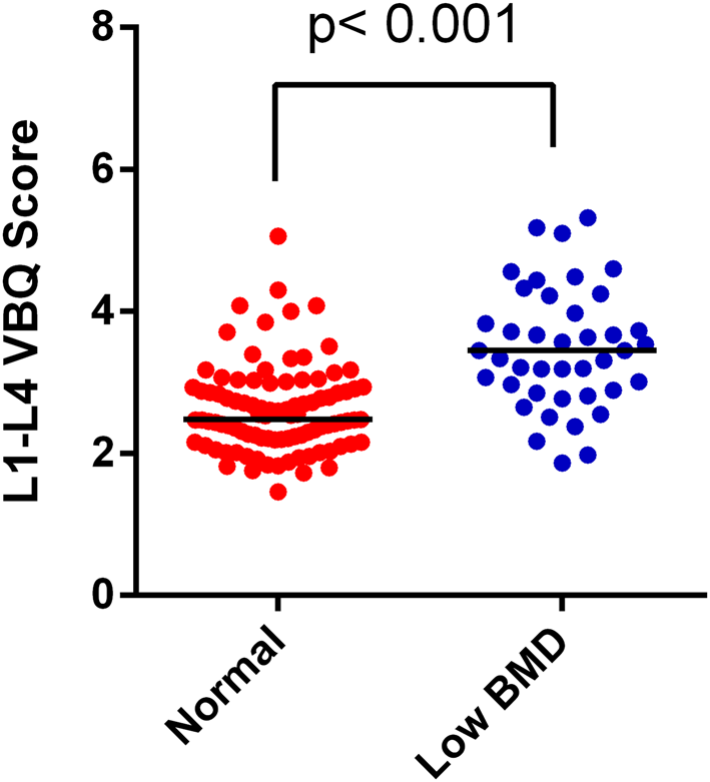
L1-L4 VBQ score differentiates between normal and low BMD in AIS patients.

A total of 136 patients were included in this study out of which 90 (66.2%) were females, and mean Cobb angle was 78.89 degree. The overall mean age was 14.84 ± 2.10 years, and the age did not differ significantly between sexes (*P* > 0.05). There was a total of 41 patients (30.1%) in the low BMD group. The detailed data are shown in Table 1. The statistically significant differences in height (*P* < 0.001), corrected height (*P* = 0.004), Cobb angle (*P* < 0.001), QCT Z-score (*P* < 0.001) and VBQ score (*P* < 0.001) were found between groups. Compared with the normal group, the low BMD group had a lower height, a larger Cobb angle and a higher VBQ score. The mean VBQ score in normal and low BMD groups was 2.62 ± 0.62 and 3.48 ± 0.85, respectively (Fig. 2). The corrected height was calculated based on a correction equation for body height by Cobb angle (corrected height = height + ∆H, ∆H = 0.6 * X + 2.6 (mm), X = ∑(Cobb-30))^18^. No significant differences in sex, age, weight, body mass index (BMI), Lenke classification and Risser sign were found between two groups (*P* > 0.05). The ICC for interrater reliability was 0.84 (95% CI 0.71–0.92) and the ICC for intra-rater reliability was 0.92 (95% CI 0.86–0.94).

**Table 1:** Demographics, MRI- and QCT-derived Measurements for the Study Population in AIS.

Variable	Entire Cohort (*n* = 136)	Normal BMD (*n* = 95,69.9%)	Low BMD (*n* = 41,30.1%)	*P*-value
Female, *n*, %	90(66.2)	64(67.4)	26(63.4)	0.695
Age, year	14.84 ± 2.10	14.91 ± 2.15	14.33 ± 1.75	0.778
Weight, kg	45.17 ± 9.31	46.00 ± 9.31	43.25 ± 9.15	0.114
Height, cm	150.82 ± 12.14	153.25 ± 12.59	145.17 ± 8.84	0.000
Corrected Height, cm	155.05 ± 10.86	156.77 ± 11.49	151.05 ± 8.06	0.004
Corrected BMI, kg/m2	18.70 ± 3.1	18.61 ± 2.81	18.90 ± 22.87	0.657
Lenke Classification, n, %				0.108
1	65(47.8)	50(52.6)	15(36.6)	
2	10(7.4)	4(4.2)	6(14.6)	
3	24(17.6)	15(15.8)	9(22.0)	
4	1(0.7)	0(0.0)	1(2.4)	
5	27(19.9)	19(20.0)	8(19.5)	
6	9(6.6)	7(7.4)	2(4.9)	
Cobb angle, degree	78.89 ± 36.81	70.87 ± 34.19	97.50 ± 36.32	0.000
Risser	3.36 ± 1.32	3.37 ± 1.26	3.17 ± 2.14	0.827
VBQ score	2.88 ± 0.80	2.62 ± 0.62	3.48 ± 0.85	0.000
QCT, mg/cm^3^	139.83 ± 50.22	164.10 ± 37.74	83.59 ± 37.74	0.000
T-score	− 1.09 ± 1.68	− 0.27 ± 1.24	− 2.99 ± 0.81	0.000
Z-score	− 0.93 ± 1.61	− 0.13 ± 1.19	− 2.79 ± 0.57	0.000

BMD bone mineral density, BMI body mass index, VBQ vertebral bone quality, QCT quantitative computed tomography, AIS adolescent idiopathic scoliosis.

### Factors related to QCT Z-score

**Figure 3:**
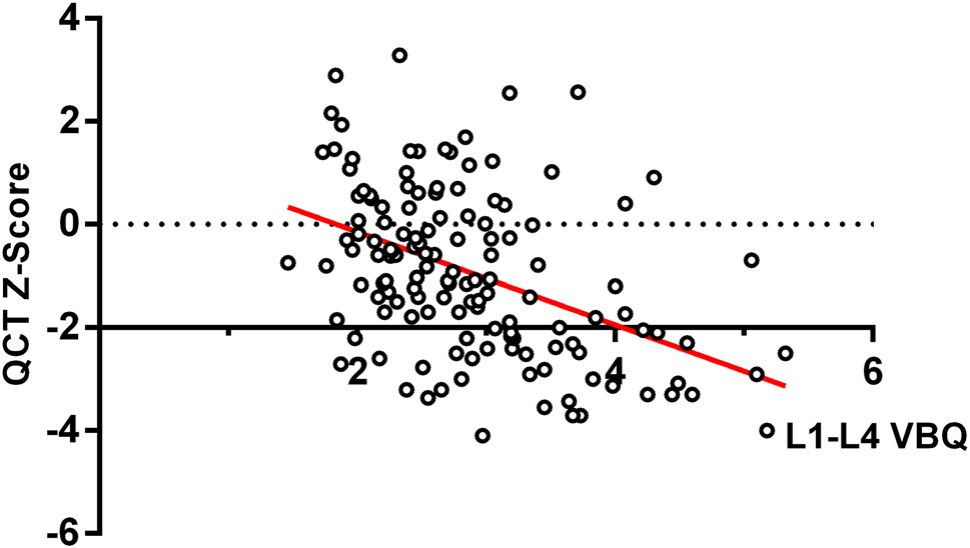
The correlation between QCT Z‑score and L1-L4 VBQ.

The QCT Z-score was significantly correlated with the Cobb angle of the major curve (*r* =  − 0.430; *P* < 0.001) and VBQ score (*r* =  − 0.454; *P* < 0.001). Figure 3 shown the correlation between QCT Z‑score and VBQ score. A multivariable logistic regression analysis was performed to adjusted for height, Cobb angle and VBQ score. The model revealed that the Cobb angle (OR: 1.014, 95% CI 1.002–1.026, *P* = 0.027) and VBQ score (OR: 4.134, 95% CI 2.136–8.000, *P* < 0.001) could independently associated with low BMD in AIS patients.

### Diagnosis performance of the VBQ score

**Figure 4:**
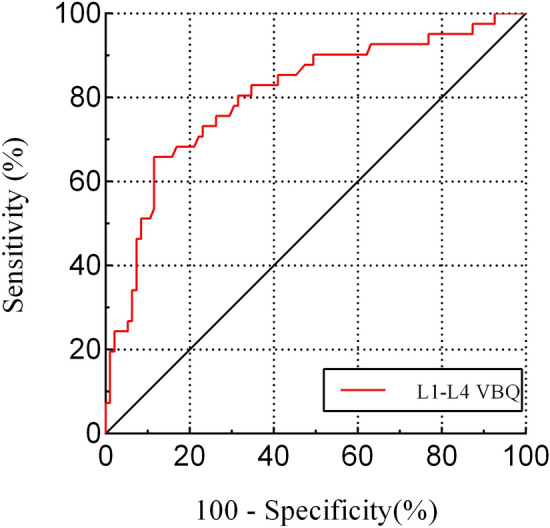
Receiver operating characteristic curve (ROC) of L1-L4 VBQ showing the sensitivity and specificity.

According to the AUC of ROC curve shown in Fig. 4, the diagnosis accuracy of the VBQ score for distinguishing low BMD was 0.806. Table 2 presents the diagnosis performance of the VBQ score with various proposed thresholds (cutoff value) in distinguishing normal from low BMD. According to the maximum Youden index, a cutoff value at 3.18 had the best diagnosis performance for distinguishing low BMD in AIS, with a sensitivity of 65.9% and a specificity of 88.4%.

**Table 2:** Diagnostic performance of L1-L4 VBQ for distinguishing low bone mineral density in AIS.

Variable	Cutoff value with high sensitivity (about 90%)	Cutoff value with high specificity (about 90%)	Cutoff value with balanced sensitivity and specificity
L1-L4 VBQ	2.49	3.42	3.18
Sensitivity (95% CI), %	90.2(76.87–97.28)	51.2(35.13–67.12)	65.9(49.41–79.92)
Specificity (95% CI), %	50.5(42.12–62.97)	91.6(85.41–96.99)	88.4(81.49–94.84)

VBQ vertebral bone quality, CI confidence intervals, AIS adolescent idiopathic scoliosis.

## Discussion

Poor bone quality was a generalized phenomenon and a systematic disorder in AIS patients, although the reason is not yet clear. In 1982, Burner et al.^19^ first reported low BMD status in patients with AIS, based on plain radiographs. Since then, several studies have corroborated these findings and the prevalence of low BMD in AIS is approximately 20–38%^20–22^. The follow-up studies indicated that low BMD in AIS patients may be a persistent phenomenon, increasing the risk of osteoporosis during adulthood^7,23^. More importantly, persistent low BMD is demonstrated to be an independent and significant risk factor for the curve severity and progression^7–9^. In our study, low BMD was found in 30.1% of patients, and the BMD derived from QCT was significantly negative correlated with the Cobb angle of the major curve, which was consistent with previous studies. Spinal fusion is the ultimate intervention for patients with AIS and its positive outcomes were influenced by the bone quality. Any of negative complications can lead to loss of alignment and surgical failure. Therefore, routine BMD screening is critical for AIS patients.

Currently, DXA has been widely used to evaluate BMD in clinical practice; however, it may not be reliable for the evaluation of BMD in patients with spinal deformities, especially scoliosis^24^. In addition, DXA is unable to take into account changes in body and skeletal size during growth, limiting its usefulness in longitudinal studies, whereas QCT can assess both volume and density of bone in the axial and appendicular skeleton, it may be therefore more useful than DXA in adolescents^25^. However, QCT cannot be a reusable tool in BMD monitoring due to its higher radiation dose. Researchers have been exploring additional tools with convenience and simplicity to assess BMD.

More recently, a novel MRI-based VBQ score was proposed as an opportunistic osteoporosis screening tool. The BMD assessment method based on MRI could assess the fatty infiltration extent of trabecular bone, thereby reflecting the pathophysiological change of bone mass abnormalities. Unlike DXA and QCT, MRI-based VBQ score was radiation free and demonstrated generalizability across different MR systems^12^. But it is noteworthy that almost all previous studies about VBQ score were of the adults. To best of our knowledge, it is the first preliminary investigation of the application of the VBQ method in assessing BMD in AIS patients. The results of our study showed significantly differences in VBQ score between BMD subgroups. The patients with low BMD had a higher VBQ score, which was consistent with previous studies^26–29^. VBQ score had a moderate to strong negative linear correlation with QCT-based BMD. In addition, a higher VBQ score had the ability to independently predict low BMD in AIS patients.

The VBQ score exhibited a good discriminability between AIS patients with and without low BMD (AUC 0.806), and a cutoff point of 3.18 was obtained for identifying low BMD, based on the maximum Youden index. Some studies have pointed that the stability of the VBQ may be compromised when abnormal spinal morphology was present. Salzmann SN et al.^28^ observed that the VBQ score had a sensitivity of 74.3%, a specificity of 57.0%, and an AUC of 0.707 to distinguish osteopenia/osteoporosis from normal BMD in patients undergoing lumbar spinal fusion. Roch PJ et al.^29^ reported a sensitivity of 74.3% and a specificity of 60.4% with an AUC of 0.713. In contrast, our reported VBQ sensitivity of 65.9% and specificity of 88.4% was no inferior to other studies. These results suggested that the VBQ score still had good diagnostic efficacy in identifying low BMD in patients with scoliosis. Based on our results, we suggest that a VBQ score ≥ 3.18 can be used to distinguish patients with low BMD. Lastly, the inter-rater and intra-rater reliability analysis showed a fair repeatability of VBQ score method, demonstrating its potential feasibility in clinical practice.

The application of the VBQ method in clinical practice has several advantages. MRI has been widely used as a standard preoperative screening before AIS surgery. It is a reliable and easy-to-use tool that enables surgeons to evaluate nerve and spinal cord conditions while bringing higher clinically opportunistic screening rates for BMD to patients in an economic-efficient and radiation-free manner, thereby reducing unnecessary radiation exposure. Besides, poor bone quality is one of the risk factors for a worsening scoliosis curve, so repeated monitoring of BMD is necessary throughout the treatment process of AIS patients. The MRI-based VBQ method is a highly reproducible due to ionizing radiation free. Finally, theoretically, the L1-L4 VBQ may have another advantage. In patients undergoing spinal surgery, it may better reflect localized bone quality in the lumbar spine and may provide a more accurate picture of bone quality in the surgical area. Although this hypothesis is theoretically sound, further studies are needed to validate it, such as comparing differences in cervical, thoracic, and lumbar VBQs.

Of note, the current study does not suggest that MRI replace DXA or QCT for the assessment of low BMD or osteoporosis. Instead, we believe that the utilization of threshold value we proposed can allow for more selective utilization of these screening methods and avoid some unnecessary radiation exposure. As a rough screening tool, VBQ score is sufficient to potentially differentiate between healthy and low BMD vertebrae with 88% specificity.

This is a preliminary exploratory study and still has some limitations. Firstly, due to its retrospective nature and relatively limited sample size, this study has some potential biases. Secondly, the AIS patients from our institution have regional characteristics and are mostly minorities. Thirdly, as the study was based on radiological findings, histopathological analysis is needed for further validation of VBQ in the future. Lastly, the signal intensity ratios of vertebral body vs cerebrospinal fluid may be influenced by the MRI vendor, field strength or other acquisition parameters, which requires further validation studies.

## Conclusion

This is first study to investigate the diagnosis value of MRI-based VBQ score in assessing BMD for AIS patients and the correlation between VBQ score and BMD derived from QCT. The results of the study indicated the novel VBQ score was a promising tool in distinguishing low BMD in patients with AIS, with a diagnostic precision of 81%. The VBQ score based on MRI would enable surgeons to preliminarily evaluate the bone density while assessing neurological and spinal cord conditions, with a value ≥ 3.18 indicating a high suspicion of low BMD, and further examination (e.g., using QCT) is recommended to clarify the diagnosis.

## Data Availability

The datasets used and/or analyzed during the current study available from the corresponding author on reasonable request.
